# Destined to Die: Apoptosis and Pediatric Cancers

**DOI:** 10.3390/cancers11111623

**Published:** 2019-10-23

**Authors:** Zhang’e Choo, Amos Hong Pheng Loh, Zhi Xiong Chen

**Affiliations:** 1 Department of Physiology, Yong Loo Lin School of Medicine, National University of Singapore, Singapore 117593, Singapore; e0225128@u.nus.edu; 2 VIVA-KKH Pediatric Brain and Solid Tumor Program, KK Women’s and Children’s Hospital, Singapore 229899, Singapore; amos.loh.h.p@singhealth.com.sg; 3 Department of Pediatric Surgery, KK Women’s and Children’s Hospital, Singapore 229899, Singapore; 4 National University Cancer Institute, Singapore, Singapore 119074, Singapore

**Keywords:** cancer, apoptosis, pediatric, development

## Abstract

Apoptosis (programmed cell death) is a systematic and coordinated cellular process that occurs in physiological and pathophysiological conditions. Sidestepping or resisting apoptosis is a distinct characteristic of human cancers including childhood malignancies. This review dissects the apoptosis pathways implicated in pediatric tumors. Understanding these pathways not only unraveled key molecules that may serve as potential targets for drug discovery, but also molecular nodes that integrate with other signaling networks involved in processes such as development. This review presents current knowledge of the complex regulatory system that governs apoptosis with respect to other processes in pediatric cancers, so that fresh insights may be derived regarding treatment resistance or for more effective treatment options.

## 1. Introduction

Apoptosis, a genetically coded programmed cell death, is a natural physiologic process evolutionarily conserved from worm to man [1]. Apoptosis occurs during embryonic development and is critical for maintaining tissue homeostasis. It also acts as a defense mechanism to eliminate unwanted cells such as those in immune reactions or cells with potentially harmful mutations damaged by disease or noxious agents [2,3]. Dysregulation of apoptosis is an important aspect of cancer pathogenesis as it disrupts the delicate balance between cell proliferation and cell death, and has been widely recognized as a hallmark of cancer [4,5].

For decades, defects in apoptosis during development have been implicated in the formation and progression of cancers including childhood malignancies [6], as many of these embryonal neoplasms and developmental processes share similar biological mechanisms. As conventional chemotherapy regimens primarily exert their anti-tumor activity by triggering the cells’ intrinsic cell death programs [7], this review provides insights on the interaction of apoptosis with other signaling pathways during pediatric cancer development, and how the apoptotic cascade can be further exploited for more targeted therapies in the treatment of these cancers. 

## 2. Apoptosis Signaling Pathways

**Figure 1:**
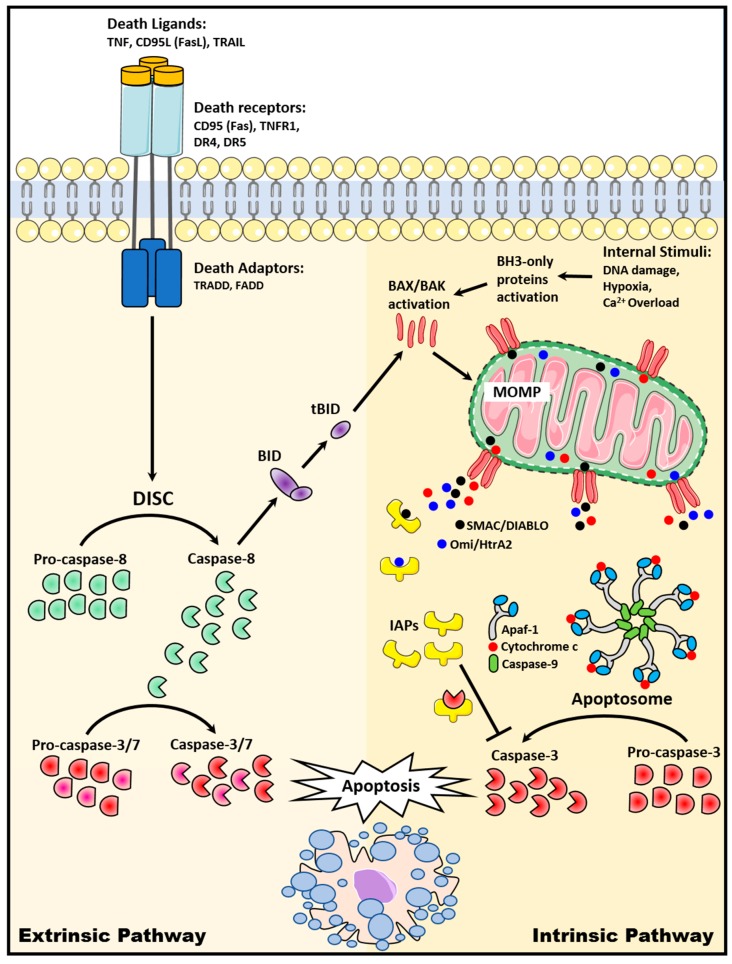
Extrinsic and intrinsic apoptosis signaling pathways.

Two major apoptosis pathways have been widely described: (1) The extrinsic apoptotic pathway, which involved signaling from cell surface death receptors and (2) the intrinsic apoptotic pathway, which involved mitochondria [8] (Figure 1).

### 2.1. The Extrinsic Death Receptor Pathway

The extrinsic apoptotic pathway is activated when death ligands, which are predominantly expressed on immune cells such as activated T lymphocytes, natural killer cells, and macrophages, bind to its death receptors (DRs) [9,10,11]. Several DRs of the tumor necrosis factor (TNF) receptor superfamily have been widely described and are ubiquitously expressed on the surface of cells. These include CD95 (Fas/APO-1), TNF receptor 1 (TNFR1), DR3 (APO-3), DR4 (TNF-related apoptosis-inducing ligand (TRAIL) receptor 1, TRAIL R1), DR5 (TRAIL R2), and DR6 [12]. These DRs contain an intracellular death domain, which is induced to recruit adaptor proteins such as Fas-associated death domain (FADD) and TNF receptor-associated death domain (TRADD) upon binding of the death ligand to its DR. A multi-protein complex, known as the death-inducing signaling complex (DISC), is subsequently formed to initiate the assembly and activation of pro-caspase-8. Activated caspase-8 then cleaves a chain of downstream caspases to execute apoptosis. [13]. Caspase-8 also cleaves BID, which then triggers the release of cytochrome c from the mitochondria and activates subsequent intrinsic apoptotic signaling [14]. 

### 2.2. The Intrinsic Mitochondrial Pathway

The intrinsic apoptotic pathway is activated when internal stimuli, such as growth factor deprivation, hypoxia, DNA damage, severe oxidative stress, and Ca^2+^ overload, are triggered within the cell [15]. BAX and BAK from the pro-apoptotic BCL-2 family of proteins are activated and form pores in the outer mitochondria membrane to trigger mitochondrial outer membrane permeabilization (MOMP). As a result, apoptogenic factors including cytochrome c, second mitochondria-derived activator of caspase/direct inhibitor of apoptosis protein-binding protein with low PI (Smac/DIABLO), apoptosis-inducing factor (AIF), and Omi/HtrA2 are released into the cytoplasm [16,17,18]. Cytoplasmic cytochrome c then interacts with Apaf-1 and caspase-9 to form apoptosome, a multiprotein complex that catalyzes effector caspase-3 activation, resulting in apoptosis [19]. Cytoplasmic Smac/DIABLO and Omi/HtrA2, on the other hand, bind to inhibitor of apoptosis proteins (IAPs) to disrupt the interaction of IAPs with caspase-3 or -9, thus releasing the caspases for subsequent activation and downstream apoptosis [17,20].

## 3. Dysregulation of Apoptosis and Apoptosis-Targeted Therapies in Childhood Cancers

Cancer is the second leading cause of death in children aged <14 years despite the advances in treatment over the years to increase the overall five-year pediatric cancer survival rate to approximately 80% [21,22]. The most common cancers in children include leukemias (acute lymphoblastic leukemia (ALL) and acute myeloid leukemia (AML)), brain and central nervous system (CNS) tumors, neuroblastoma, Wilms’ tumor, lymphomas (non-Hodgkin lymphomas (NHL)), rhabdomyosarcoma, and bone cancers (osteosarcoma and Ewing’s sarcoma) [23].

**Figure 2:**
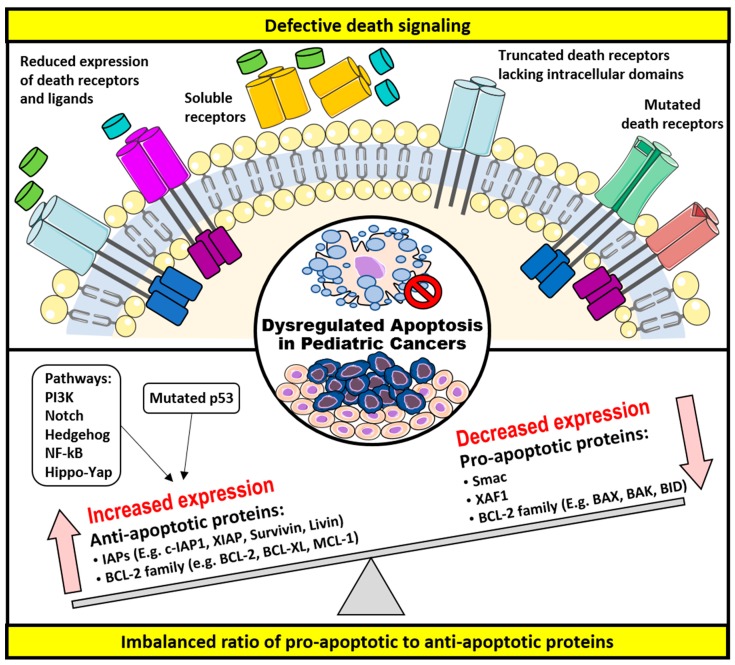
Mechanisms of apoptotic evasion in pediatric cancers and therapeutic targets.

Often, in pediatric oncology, it is not uncommon that many of the defects arise in the developmental signaling pathways such as Wnt, Hedgehog, Notch, and Hippo, all of which regulate cell fate, proliferation, migration, differentiation, apoptosis, and formation of organs/tissues [24,25,26,27]. These developmental defects at the embryonal level resulted in the accumulation of undifferentiated cells and the failure to actively remove these cells via apoptosis often predispose one to pediatric cancer development. In this review, two underlying mechanisms— (1) defective death receptor signaling and (2) imbalanced ratio of pro-apoptotic to anti-apoptotic proteins, both of which renders impaired apoptosis or development of resistance to it—will be discussed. Figure 2 summarizes the underlying the mechanisms that allow cells to escape from apoptosis in pediatric cancers and the therapeutic targets, which can reverse these mechanisms to restore apoptosis. 

### 3.1. Defective Death Receptor Signaling

Different abnormalities in the death signaling pathways have been reported to cause the evasion of the extrinsic apoptotic pathway. As described in the following, these include decreased expressions of death receptors and the ligands, as well as loss-of-function of death receptors, all of which perturbed the transmission of death signaling downstream and hence a reduced apoptosis [28,29,30,31,32,33,34,35,36,37,38,39,40,41,42,43,44,45,46,47,48].

CD95/Fas signaling, one of the most widely studied death receptor signaling, plays a significant role in immune surveillance and normal lymphocytes/melanocytes homeostasis [28]. In fact, impaired CD95 signaling has been implicated in many pediatric haemopoietic malignancies, including childhood acute leukemias (ALL and AML) and B-cell non-Hodgkin lymphomas (NHLs) [29,30,31]. Reduced expression of CD95 and its ligand CD95-L were also found to play a role in various pediatric cancers such as T-cell ALL, treatment-resistant leukemia, or neuroblastoma cells [32,33,34]. Improved prognosis and chemotherapeutic response were observed in B-cell lymphomas and AML with higher CD95 expression, respectively [35,36]. Several cytotoxic drugs such as doxorubicin, methotrexate, cytarabine, etoposide, and cisplatin are used in treatment of leukemias. These drugs are able to induce the expression of CD95 and CD95-L, which subsequently increase the cancer cells’ sensitivity to CD95 death signals or provoke the cells’ intrinsic apoptosis capability [37]. However, despite therapeutic strategies to restore death signaling in cancer cells, most primary T-cell leukemias have been reported to be constitutively resistant against CD95-induced apoptosis [38,39,40]. The resistance against CD95-induced apoptosis can be attributed to the occurrence of alternative splicing in the tumor, which produces soluble CD95 (sCD95). sCD95 function by sequestering CD95 ligand to cell surface CD95 receptor, thus impairing downstream death signaling and causing resistance against cytotoxic drug-induced, CD95-mediated apoptosis [41]. In separate reports, sCD95 was detected in several human T-cell leukemia cell lines and primary leukemic cells from infants with ALL [42,43]. Increased expression of sCD95 was also observed in patients with lymphoid and not myeloid leukemias [44]. Moreover, a splicing variant of CD95, which resulted in a truncated receptor without its intracytoplasmic death-signaling domain, also provided resistance to CD95-mediated apoptosis [45]. Altered expression as a result from polymorphisms and deleterious mutations in the CD95 promoter region were identified in childhood T-cell ALL, AML, and NHL [46,47,48].

Fortunately, with the discovery and understanding of other death signaling pathways, various therapeutic strategies have been developed to activate the extrinsic apoptotic pathway. In addition to CD95-L, there are other TNF family members (e.g., TNF-α and TRAIL), which can directly trigger apoptosis with their respective receptors (TNFR1, TRAIL R1, and TRAIL R2). TNF is a proinflammatory cytokine that activates and elicits the effects of NF-κB and caspases, thus rendering it ineffective to be used as a therapeutic target for cancer treatment [49]. In contrast to the liver toxicity caused by CD95 antibodies, recombinant TRAIL ligands and specific antibodies are well-tolerated in mouse tumor xenografts [50,51]. Despite the success of using recombinant TRAIL ligand to trigger apoptosis in several pediatric cancers including leukemia, neuroblastoma, rhabdomyosarcoma, and Ewing’s sarcoma [52,53,54,55,56], numerous cases of TRAIL-mediated resistance have also been reported. For instance, in neuroblastoma, downregulation of TRAIL receptors as a result of promoter hypermethylation was shown to decrease the response and sensitivity to TRAIL ligand, thus weakening the downstream apoptosis signaling [57,58]. Hypermethylation or genomic deletions in CASP8 gene were also identified in neuroblastoma. This resulted in the loss or reduced expression of caspase-8, an effector caspase, which mediates the downstream death signaling, and further rendering neuroblastoma resistant to TRAIL-induced apoptosis [59,60,61]. Together, these imply the complexity of exploiting the death receptor pathway. Different approaches were used combinatorically with TRAIL to improve the sensitivity and response towards TRAIL-induced apoptosis. For example, in childhood ALL, XIAP inhibitors act synergistically with TRAIL in vitro and in vivo to enhance downstream apoptotic signaling. In high BCL-2-expressing acute leukemia cells with resistance to TRAIL, XIAP inhibitors were able to induce cleavage of BCL-2 proteins and promote MOMP through conformational change of BAK, further enhancing TRAIL-induced apoptosis [62].

In addition to the use of recombinant TRAIL ligand, monoclonal antibodies against TRAIL receptors, TRAIL R1 or TRAIL R2, were developed and evaluated in preclinical and clinical settings [63,64]. For instance, Mapatumumab (HGS-ETR1), a human monoclonal antibody that specifically targets TRAIL-R1, was evaluated in vitro and in vivo by the pediatric preclinical testing program [65]. While limited activity of Mapatumumab was demonstrated against a panel of pediatric cancer cell lines in vitro (e.g., leukemia, medulloblastoma, glioblastoma, neuroblastoma, Wilms’ tumor, rhabdomyosarcoma, osteosarcoma, and Ewing’s sarcoma), significant event-free survival (EFS) was only observed in Mapatumumab-treated patient-derived xenografts of glioblastoma, neuroblastoma, and osteosarcoma origins [65]. Lexatumumab (HGS-ETR2), a human monoclonal antibody against TRAIL-R2, was also recently evaluated in a pediatric Phase I trial in children with solid tumors including Ewing’s sarcoma, osteosarcoma, neuroblastoma, and rhabdomyosarcoma [66].

### 3.2. Imbalanced Ratio of Pro-Apoptotic to Anti-Apoptotic Proteins

The intrinsic apoptotic pathway is directly regulated by two groups of proteins, the BCL-2 family and the IAPs, both of which have been widely described to exert pro- or anti-apoptotic activity in the cell [67]. Of note, the initiation of an apoptotic response was dictated by the ratio of pro- to anti-apoptotic proteins and not the absolute quantities [68]. The expression of these apoptotic proteins is also influenced by many other survival signaling pathways such as the p53, STAT, and PI3K, which indirectly affect apoptosis [69,70,71]. Often, a disrupted balance as a result of reduced expression of pro-apoptotic proteins and increased expression of anti-apoptotic proteins contributes to tumorigenesis by apoptosis evasion in cancer cells. Therefore, it is an attractive option to target these proteins as a treatment for childhood cancers.

#### 3.2.1. The BCL-2 Family of Proteins

The BCL-2 family of proteins plays a pivotal role in the regulation of apoptosis, especially in the intrinsic pathway as BCL-2 family are usually localized at or near the mitochondria to facilitate or inhibit the activation of MOMP and subsequent cell death [67]. The BCL-2 family members can be categorized according to their functions and the presence of either one or multiple BCL-2 homology [72] domains: (1) Anti-apoptotic proteins with four BH domains (e.g., BCL-2, BCL-X_L,_ BCL-w, and MCL-1), (2) pro-apoptotic proteins with four BH domains (e.g., BAX and BAK), and (3) pro-apoptotic proteins with only BH3-domain (e.g., BID, BIM, BAD, BIK, NOXA, and PUMA) [18]. BH3-only proteins are the sensors for apoptosis, which are triggered in response to cellular stresses such as growth factor withdrawal, DNA damage, and Ca^2+^ overload. They then trigger the activation and oligomerization of multi-BH-domain, BAX and BAK, which promote MOMP and apoptosis [18].

The chromosomal analysis from comparative genomic hybridization (CGH) of the BCL-2 family members identified frequent deletions of pro-apoptotic BAK, BID, and BAD loci, and overexpression of anti-apoptotic BCL-2 and BCL-XL in several tumors including childhood cancers—glioblastoma and acute leukemias [68]. In AML patients, high expression of BCL-2 was positively correlated with low disease remission rate while low expression of BCL-2 was associated with a favorable karyotypic t(8;21) translocation [73,74]. High levels of BCL-2 proteins were further observed in separate studies of childhood ALL [75,76]. Furthermore, a high BCL-2/BAX ratio was predicted to have a poorer overall survival in AML patients and be associated with relapse in childhood ALL [77,78]. Overexpression of BCL-2 was also commonly identified in many childhood solid tumors such as osteosarcoma [79], glioblastoma multiforme [80], Wilms’ tumor [81], and neuroblastoma [82], highlighting a significant BCL-2 involvement in the development of these cancers. Moreover, BCL-2 overexpression is reported to be an important mechanism by which apoptosis and drug resistance is altered in retinoic acid (RA)-induced differentiation of neuroblastoma cells [83]. In clinical practice, 13-cisRA (isotretinoin) is a well-established component of maintenance treatment for high-risk neuroblastoma. BCL-2 protein levels varied during differentiation of neuroblastoma cells, with well-differentiated subtypes containing higher BCL-2 expression [84]. Hence, combinatorial strategies of inducing differentiation in poorly differentiated neuroblastoma cells with RA, followed by pharmacologically altering BCL-2 expression could be useful to sensitize neuroblastoma to apoptosis.

In addition to alterations of the genetic loci of the BCL-2 family proteins, other mutations of genes and/or signaling pathways that can indirectly influence the expression levels of these BCL-2 family proteins were also identified in childhood malignancies. For example, p53, one of the best-known tumor suppressors, is a transcription factor that induces the expression of pro-apoptotic BAX protein [71,85]. Therefore, defects in the p53 tumor suppressor gene will lead to downregulation of BAX, hence inhibiting apoptosis. Overexpressing p53 through adenoviral-mediated transduction in childhood sarcomas (rhabdomyosarcoma, osteosarcoma, and Ewing’s sarcoma) has been observed to sensitize cells to chemotherapeutics [86,87,88]. Other than BAX, another BH3-only protein BIM was also found to be induced by the activation of p38/JNK-MAPK signaling, thus triggering apoptosis [89]. Although no direct evidence exists to link MAPK pathway in the pathophysiology of pediatric cancers, its activation or inhibition has been shown to improve drug sensitivity in these cancers. For instance, glucocorticoids (GC), which are commonly used for treatment of leukemias or lymphomas, trigger apoptosis through increased BIM expression following the activation of p38 MAPK [90]. In contrast, the inhibition of ERK1/2 MAPK in osteosarcoma cells prevented the paradoxical effect of doxorubicin-induced expression of BCL-2 and BCL-XL, thereby improving the sensitivity of doxorubicin-killing in these cells [91]. PI3K pathway, a key regulator of normal cellular processes involved in cell survival and growth, is another example of an indirect regulator of apoptosis. Through the phosphorylation by kinases in this pathway, the BCL-2 family of proteins such as BAD or BCL-2 can be inactivated or activated to inhibit or promote apoptosis, respectively [70]. Moreover, molecules such as substance P, which are often overexpressed in neuroblastoma and childhood leukemias, act upstream of and activate MAPK and PI3K pathways to inhibit apoptosis [92,93,94,95]. Inhibition of PI3K and its downstream signaling was observed to enhance apoptosis in Ewing’s sarcoma, while treatment of PI3K inhibitors in neuroblastoma cells was found to target anti-apoptotic BCL-2 family proteins and further promote mitochondrial apoptosis through chemosensitization, thus emphasizing the association between PI3K and BCL-2 family-mediated apoptosis [96,97]. Furthermore, activation of hedgehog signaling, which governs cell growth and differentiation in a wide variety of tissues during embryonic development, has been identified in several pediatric malignancies including rhabdomyosarcoma, medulloblastoma, neuroblastoma, and Wilms’ tumor [98,99,100,101]. It is worth noting that Gli1, a transactivator downstream of Hh signaling, was found to upregulate the expression of BCL-2 [102,103]. However, whether the activation of Hh signaling with increased BCL-2 expression contributed to these pediatric tumors remains to be investigated. Similarly, Notch signaling was also found to contribute to oncogenesis in children. Notch pathway has been reported to regulate numerous downstream targets, most of which with oncogenic roles such as PI3K, p53, NF-κB, BCL-2, and IAPs [26]. Therefore, activation of Notch signaling either through mutations or amplification of Notch such as those identified in T-cell ALL, medulloblastoma, osteosarcoma, and Ewing’s sarcoma, ultimately promotes cell survival and inhibits apoptosis [104,105,106,107]. MicroRNAs have also been found to regulate the levels of BCL-2 family proteins. BCL-2 protein can be targeted by several microRNAs such as miR-143, whereby an overexpression of miR-143 in osteosarcoma promotes apoptosis by downregulating BCL-2 [108]. In rhabdomyosarcoma, STAT3 was observed to be constitutively activated to induce the expression of several target genes including anti-apoptotic BCL-2, BCL-XL, and Survivin, while transcription factors such as PAX3 and PAX3/FKHR were shown to induce BCL-XL mRNA expression, thus highlighting the involvement of BCL-2 family proteins in rhabdomyosarcoma tumorigenesis [69,109]. Accordingly, treatment with STAT3 inhibitor, XZH-5, caused suppression of BCL-2, BCL-XL, and Survivin, and resulted in increased apoptosis in rhabdomyosarcoma cells [110]. Taken together, the complexity surrounding the regulation of apoptosis conferred either directly by BCL-2 family proteins and IAPs or indirectly by several survival pathways expands the range of therapeutics strategies available to trigger apoptosis in pediatric cancers.

Since high expression of anti-apoptotic BCL-2 family proteins has been widely reported and highly associated with resistance towards chemotherapy and TRAIL in various pediatric cancers such as leukemias, rhabdomyosarcoma, and neuroblastoma [111,112,113,114,115], several approaches have been proposed over the years to inhibit the function these proteins. For instance, ABT-737, a BH3 mimetic that selectively targets BCL-2, BCL-XL, and BCL-w, inhibits the activity of these pro-apoptotic proteins and releases BAX and BAK from their interaction, thus allowing the formation of pores in the outer mitochondrial membrane and activating apoptosis [116]. As ABT-737 selectively targets certain anti-apoptotic proteins, a method called BH3 profiling was developed to detect and analyze cancer cells’ dependency on BCL-2 and to predict the sensitivity of ABT-737 in these cells. Using this method, ALL primary and commercial cell lines were predicted to be responsive to ABT-737 treatment [117]. Indeed, in two separate studies conducted in childhood ALL, ABT-737 was shown to be effective as a single agent against pediatric ALL xenografts as well as synergistically potentiate the cytotoxic effects of clinically available chemotherapy drugs (e.g., topotecan, dexamethasone, etoposide) in both ALL cell lines and xenografts [118,119]. Similarly, BH3 profiling was used to stratify neuroblastomas with a specific type of BCL-2 family-mediated resistance and thus facilitate in the prediction of neuroblastomas’ sensitivity towards different BCL-2 inhibitors. The release of cytochrome c was measured as an output of BH3 profiling after different BH3-domain peptides were added to stimulate the mitochondria extracted from neuroblastoma cell lines. Three different subsets of neuroblastomas with different BCL-2 family proteins’ dependency were identified: Those that were (1) high in MCL-1 expression (reacted with NOXA-BH3 peptides and are sensitive to killing by MCL-1 inhibitor AT-101 and not ABT-737), (2) high in both/either BCL-XL or BCL-w expression (reacted with BIK-BH3 peptides and are sensitive to killing by ABT-737), and (3) relapsed cases, which are insensitive to both BH3 peptides and inhibitors [72]. Consistently, in another study, ABT-737 showed single-agent activity against only BIM:BCL-2 and not BIM:MCL-1-primed neuroblastoma-derived xenografts [120]. ABT-737 has a low binding affinity towards MCL-1 and several resistance cases of ABT-737 as a result of high MCL-1 expression have been described not only in neuroblastoma, but also in other cancers [121,122]. The silencing of MCL-1 was shown to sensitize or prime cancer cells to cytotoxic chemotherapy and BCL-2 family antagonists in ALL, rhabdomyosarcoma, and neuroblastoma [123,124,125,126].

Collectively, these findings suggest that combinatorial targeting of different components of the mitochondrial apoptotic pathway may be useful to overcome resistance and improve treatment outcome. Intriguingly, despite the development of another BH3 mimetic Obatoclax (GX15-070), which can also target MCL-1 in addition to BCL-2 and BCL-XL, and with its efficacy tested in various adult cancers [127], its effectiveness in childhood cancers was, nevertheless, not well-studied. Furthermore, even though ABT-737 performs efficiently as a single agent or combinatorically with cytotoxic drugs against AML [128,129] and lymphoma [130] cell lines, it has a poor oral bioavailability, which limits its usage in clinical setting. Therefore, a derivative, ABT-263 (navitoclax), which is orally bioavailable and with similar activity, was developed and evaluated in a pediatric preclinical testing program. [131]. The sensitivity of ABT-263 was tested in the program across a panel of 23 pediatric cancer cell lines and 44 mice xenografts. Of note, ABT-263 was most potent against ALL cell lines and xenografts with complete remissions in 50% (3/6) of ALL mouse xenografts [132]. Consistent with the above results, a separate study demonstrated a potent anti-tumorigenic activity of ABT-263 against different subtypes of pediatric ALL [133]. Collectively, these findings accelerated the clinical development of ABT-263, which is currently evaluated in Phase I clinical trial in combination with ABT-199 (Venetoclax) for children with relapsed/refractory ALL or lymphoblastic lymphoma (ClinicalTrials.gov Identifier: NCT03181126). There are also other several BH3 mimetics (e.g., TW-37, 73R) that were developed over the years. However, limited studies were available to investigate their efficacy against pediatric cancers [134,135].

In addition to BCL-2 family inhibitors, RNAi or antisense therapies are alternative strategies to antagonize anti-apoptotic BCL-2 family proteins. Several antisense oligonucleotides have been reported, but only Genasense (G3139) has been evaluated in Phase I clinical trial in combination with doxorubicin and cyclophosphamide for children with relapsed solid tumors (ClinicalTrials.gov Identifier: NCT00039481) [136,137]. Taken together, these results discussed the multiple strategies to target BCL-2 family proteins of the intrinsic mitochondrial apoptotic pathway and present new windows and opportunities for future therapeutic options in childhood cancers.

#### 3.2.2. The IAPs

The IAPs are a family of eight structurally and functionally similar proteins characterized by the presence of baculoviral IAP repeat (BIR) domain. The IAPs include NAIP (BIRC1), c-IAP1 (BIRC2), c-IAP2 (BIRC3), X-linked IAP (XIAP, BIRC4), Survivin (BIRC5), Apollon (BRUCE, BIRC6), Livin/ML-IAP (BIRC7), and IAP-like protein 2 (ILP-2, BIRC8) [138]. The IAPs function by interacting directly with the caspases via their conserved BIR domains, thus inhibiting caspase activity and triggering caspases degradation [139]. Moreover, these IAPs can directly interact with pro-apoptotic proteins that are involved in the apoptotic pathway. Hence, together these IAPs help to moderate the extent of apoptosis within the signaling cascade. Therefore, the increase in expression of IAPs as a result of direct genetic alterations in IAPs’ loci or the decrease in pro-apoptotic proteins to inhibit IAPs can impair apoptotic signaling to favor cell survival and contribute to cancer development.

XIAP, the most potent anti-apoptotic IAP, blocks apoptosis by inhibiting the activation of caspase-3, -7, and -9 and its dysregulation is commonly found in pediatric cancers [138]. For instance, in two independent studies, XIAP is overexpressed in childhood AML associated with poor prognosis. The high XIAP level is correlated with immature blast phenotype and cytogenetic profiles of high-risk groups, reduced relapse-free survival, and poor treatment response to induction chemotherapy [140,141]. Similarly, high XIAP expression is found in childhood T-cell ALL and its expression is associated with poor prednisone treatment response [142]. As the association was seen correlated to the levels of XIAP protein and not its mRNA, this highlights the presence of possible post-transcriptional or post-translational regulation on XIAP [142]. Indeed, an internal ribosomal entry site (IRES) element was found residing in XIAP mRNA to promote internal translation initiation in spite of conditions that terminate protein synthesis [143]. An example of a likely outcome of this is the implication of XIAP in mediating resistance to cell death induction by TRAIL in various resistant leukemia cell lines [144].

Survivin, the smallest member of IAPs with only one BIR domain, plays a pivotal role in mitosis regulation in addition to apoptosis [145]. It is involved in cytokinesis during early embryogenesis and is strongly expressed ubiquitously throughout embryonic development (e.g., thymocytes and brain development) [146,147]. As Survivin expression is found mainly in embryonic tissues and not adult tissues, it is particularly relevant to pediatric oncology [148]. Moreover, Survivin is a target gene of Yap, a core transcriptional co-activator in Hippo signaling pathway that is highly involved in embryonic organ development [149]. Intriguingly, dysregulation of Hippo pathway with overexpression of Yap has been reported in several pediatric cancers whereby Survivin is also overexpressed, suggesting possible implication of Hippo-Survivin signaling in these cancers [150,151,152,153]. Overexpression of Survivin is commonly found in pediatric cancers and has a correlation with the progression of disease, chemoresistance, metastasis, unfavorable prognosis, and survival. Such findings have been found in independent studies on AML, ALL, neuroblastoma, Wilms’ tumor, osteosarcoma, rhabdomyosarcoma, and Ewing’s sarcoma [154,155,156,157,158,159,160]. For example, Survivin gene maps to chromosome 17q25, a region that is frequently amplified in neuroblastoma [156]. Several risk factors for neuroblastoma have been identified to be significantly correlated with high Survivin expression and these include poor prognosis, reduced expression of TrkA, later age of onset, and advanced cancer stage [161,162,163,164]. Furthermore, other than Survivin itself, the Survivin gene locus also encodes for multiple genetic splice variants, which have unique properties and functions. These splice variants can also be dysregulated and play a role in tumorigenesis [165]. For instance, in childhood de novo AML, an increased ratio of splice variants, Survivin-2B/ΔEx2, was found to be associated with poorer survival outcome and treatment-resistant cases [154]. Intriguingly, Survivin-2B, one of the Survivin splice variants, has a pro-apoptotic function despite the canonical anti-apoptotic function of Survivin. For instance, low expression of Survivin-2B was found in pediatric precursor B-cell ALL and was associated with increased risk of early relapse and high-risk group [166]. Consistently with its pro-apoptotic role, low expression of Survivin-2B was also observed in some clinically unfavorable neuroblastomas while it is expressed at higher levels in clinically favorable tumors [156]. While most findings suggest poor prognosis with high Survivin expression in cancers, a study conducted on osteosarcoma conversely showed that prolonged survival was significantly associated with Survivin that was found in the nucleus and not cytoplasm [167]. Therefore, taken together, these findings demonstrate not only the role of Survivin in cancers, but also the complexity of response to Survivin in terms of its expression, localization in cells, and splice variants, which can be exploited as a useful tool for risk stratification, prognosis, as well as treatment evaluation in childhood cancers.

In addition to XIAP and Survivin, other IAPs were also shown to be involved in pediatric malignancies. For instance, high Apollon expression found in childhood AML was associated with unfavorable three-year relapse-free survival and day-7-induction chemotherapy response [168]. Interestingly, this is the first study that reported prognostic relevance of Apollon expression in human malignancies. Similarly, in a more recent study, it was shown that Apollon overexpression found in AML and ALL pediatric patients was significantly associated with poor prognosis with shorter overall and disease-free survival [169]. Another IAP, Livin, was also found to be overexpressed in osteosarcoma and its nuclear expression was significantly correlated with decreased overall survival [79]. However, in two independent studies on pediatric ALL, a high level of Livin was shown to correlate with favorable rather than unfavorable prognosis [170,171]. Although the exact mechanism was not identified, Livin has been described in another study to exhibit pro-apoptotic activity. It was reported that the truncated form of Livin, generated upon its cleavage by caspases, promotes apoptosis [172].

Moreover, there are also pro-apoptotic proteins involved in pediatric cancers through their interaction and disruption of IAPs’ activity. Examples include Smac and XAF1 (XIAP-associated factor 1). Smac is an endogenous pro-apoptotic protein of the mitochondria, which is released during intrinsic apoptotic signaling. It inhibits the activity of IAPs such as XIAP, c-IAP1, and c-IAP2 either through direct binding to IAPs, thus interfering IAPs-caspase interactions, or promotes proteasome-mediated degradation of IAPs, both of which induces apoptosis [173]. High expression of Smac has been reported to be significantly associated with two promising clinical outcomes in AML patients—improved overall survival and complete remission rate, whereas low Smac level correlated with poor karyotype [174]. Furthermore, Smac overexpression was used to sensitize osteosarcoma cells to cytotoxic drug-induced apoptosis [175]. XAF1, on the other hand, is a potent antagonist of anti-apoptotic XIAP and Survivin [176]. XAF1 was reported to be upregulated during nerve growth factor (NGF)-withdrawal pathway, an apoptotic pathway that occurs during the neural crest development of sympathetic nervous system [177]. During development, NGF is synthesized and required for maintaining the survival and differentiation of neuronal progenitors. Neuronal progenitors that are locally deprived of NGF, on the other hand, were shown to undergo intrinsic mitochondrial apoptosis. [178,179,180,181]. Failure of this process has been shown to be implicated in pediatric malignancies including neuroblastoma [182,183]. Loss of XAF1 expression was observed in neuroblastoma and was associated with poor survival and disease status [184].

With the identification of key apoptotic regulators in childhood cancers, different strategies to oppose the anti-apoptotic effects of IAPs have been developed to restore death signaling. For example, Smac mimetics and peptides were developed to target XIAP and c-IAPs. Smac mimetics interfere with the XIAP-dependent inhibition of caspases and promote the auto-ubiquitylation of c-IAP1 and c-IAP2, thus facilitating their degradation via proteasome. This in turn induces the NF-κB signaling and the production of TNFα, resulting in the activation of caspase-8 and downstream apoptosis [185,186]. Smac mimetics were reported to enhance TRAIL-or chemotherapy-mediated apoptosis in glioblastoma and neuroblastoma [187,188,189]. An overexpression of Smac has also been shown to enhance the sensitivity of neuroblastoma and glioblastoma cells to radiation treatment with increased mitochondrial perturbation and caspases activation [190]. Furthermore, an increased Smac expression was observed to inhibit the growth of neuroblastoma cells by promoting apoptosis and suppressing the rate of migration and proliferation [191]. In a more recent study, Smac mimetic LCL161, was evaluated as a single agent in a pediatric preclinical testing program. While LCL161 was found to be potent against several pediatric leukemia and lymphoma cell lines in vitro, LCL161 induced significant differences in event-free survival distribution only in one-third of solid tumor xenografts, such as osteosarcoma and glioblastoma, but not ALL xenografts [192]. On the contrary, another Smac mimetic BV6 was reported to be potent in vitro and in vivo as a single agent, as well as enhancing the efficacy of conventional induction chemotherapy including vincristine, dexamethasone, and asparaginase, leading to prolonged remission in pediatric ALL [193]. BV6 was also shown to cooperate with demethylating agents to induce cell death in ALL [194]. Together, these findings suggest that there is varying potency among different derivatives of Smac mimetic, further increasing the complexity of apoptosis-based therapies in childhood cancers.

In addition to Smac mimetic, XIAP can also be targeted using antisense oligonucleotides (ASO), which was demonstrated to be potent against several cancers either as a single agent or in combination with conventional chemotherapeutics [195,196]. XIAP ASO, AEG35156, was reported to suppress XIAP expression and induce apoptosis in several pediatric cancer cell lines including osteosarcoma, neuroblastoma, rhabdomyosarcoma, and Ewing’s sarcoma cells. AEG35156 also helps in sensitizing these cells to clinically relevant cytotoxic agents [197]. Moreover, the effect of AEG35156 has also been evaluated in early Phase I/II clinical trials in combination with chemotherapy in AML patients. A better outcome in patients with refractory AML was observed as compared to single induction regimen [198]. Despite the findings in adult AML cases, it may be a promising approach as an apoptosis-based therapy in childhood leukemias, though not yet tested in the clinic in children. Of note, XIAP inhibitors were also shown to enhance TRAIL-, CD95-, or chemotherapy-induced apoptosis in childhood ALL in vitro and in vivo [62,199,200]. Similarly, in AML, these XIAP inhibitors were found to suppress cell clonogenicity, promote cell differentiation, and synergistically enhance TRAIL-mediated apoptosis [201,202].

In addition to Smac mimetics and XIAP inhibitors, inhibitors targeting Survivin have been widely described and tested in childhood cancers. For instance, osteosarcoma cells treated with small molecule inhibitor YM155 potently reduced the expression of Survivin, suppressed cell growth, and induced apoptosis [203]. Survivin ASO, on the other hand, was shown to act synergistically with TRAIL to trigger apoptosis in neuroblastoma and also promote caspase-dependent and -independent signaling, resulting in cell death [204,205,206]. Furthermore, a Survivin minigene DNA vaccine was developed and was tested to be effective in suppressing the spread and growth of tumor in a syngeneic mouse model of neuroblastoma [207]. Taken together, several strategies have been presented to target intrinsic mitochondrial apoptotic pathway in pediatric cancers either through direct antagonizing of anti-apoptotic IAPs or act synergistically with conventional chemotherapeutics.

## 4. Conclusions

Understanding the apoptotic processes over the years has led to the advancement of new therapeutic strategies in the treatment for different cancers. Various approaches in attempting to induce apoptosis in pediatric cancers have been developed—cancers that evolved mainly during the early developmental stages due to the impairment of apoptosis for physiological culling during cell maturation and differentiation. Molecular targeted therapies that perturb developmental pathways dysregulated in the tumor may have devastating effects on normal tissues in a developmental stage and tissue-specific manner. Therefore, therapies centered around apoptosis remain as one of the most promising strategies for treating childhood malignancies. Future studies need to emphasize the identification of predictive biomarkers and rational design of combination therapies in cancer to better stratify patients who will achieve best clinical outcomes from these therapies. 

## References

[1] Hengartner M.O. (2000). The biochemistry of apoptosis. Nature.

[2] Norbury C.J., Hickson I.D. (2001). Cellular responses to DNA damage. Annu. Rev. Pharmacol. Toxicol..

[3] Thompson C.B. (1995). Apoptosis in the pathogenesis and treatment of disease. Science.

[4] Danial N.N., Korsmeyer S.J. (2004). Cell death: Critical control points. Cell.

[5] Hanahan D., Weinberg R.A. (2011). Hallmarks of cancer: The next generation. Cell.

[6] Evan G.I., Vousden K.H. (2001). Proliferation, cell cycle and apoptosis in cancer. Nature.

[7] Makin G. (2002). Targeting apoptosis in cancer chemotherapy. Expert Opin. Ther. Targets.

[8] Wong R.S. (2011). Apoptosis in cancer: From pathogenesis to treatment. J. Exp. Clin. Cancer Res..

[9] Nagata S. (1997). Apoptosis by death factor. Cell.

[10] Falschlehner C., Schaefer U., Walczak H. (2009). Following TRAIL’s path in the immune system. Immunology.

[11] Sedger L.M., McDermott M.F. (2014). TNF and TNF-receptors: From mediators of cell death and inflammation to therapeutic giants—Past, present and future. Cytokine Growth Factor Rev..

[12] Locksley R.M., Killeen N., Lenardo M.J. (2001). The TNF and TNF receptor superfamilies: Integrating mammalian biology. Cell.

[13] Wallach D., Varfolomeev E.E., Malinin N.L., Goltsev Y.V., Kovalenko A.V., Boldin M.P. (1999). Tumor necrosis factor receptor and Fas signaling mechanisms. Annu. Rev. Immunol..

[14] Walczak H., Krammer P.H. (2000). The CD95 (APO-1/Fas) and the TRAIL (APO-2L) apoptosis systems. Exp. Cell Res..

[15] Tait S.W., Green D.R. (2010). Mitochondria and cell death: Outer membrane permeabilization and beyond. Nat. Rev. Mol. Cell Biol..

[16] Kroemer G., Galluzzi L., Brenner C. (2007). Mitochondrial membrane permeabilization in cell death. Physiol. Rev..

[17] Saelens X., Festjens N., Vande Walle L., van Gurp M., van Loo G., Vandenabeele P. (2004). Toxic proteins released from mitochondria in cell death. Oncogene.

[18] Shamas-Din A., Kale J., Leber B., Andrews D.W. (2013). Mechanisms of action of Bcl-2 family proteins. Cold Spring Harb. Perspect. Biol..

[19] Riedl S.J., Salvesen G.S. (2007). The apoptosome: Signalling platform of cell death. Nat. Rev. Mol. Cell Biol..

[20] Vande Walle L., Lamkanfi M., Vandenabeele P. (2008). The mitochondrial serine protease HtrA2/Omi: An overview. Cell Death Differ..

[21] Murphy S.L., Xu J., Kochanek K.D. (2013). Deaths: Final data for 2010. Natl. Vital Stat. Rep..

[22] Howlader N.N.A., Krapcho M., Garshell J., Neyman N., Altekruse S.F., Kosary C.L., Yu M., Ruhl J., Tatalovich Z., Cho H. SEER Cancer Statistics Review, 1975–2010. https://seer.cancer.gov/archive/csr/1975_2010/.

[23] Ward E., De Santis C., Robbins A., Kohler B., Jemal A. (2014). Childhood and adolescent cancer statistics, 2014. CA Cancer J. Clin..

[24] Koesters R., Von Knebel Doeberitz M. (2003). The Wnt signaling pathway in solid childhood tumors. Cancer Lett..

[25] Oue T., Yoneda A., Uehara S., Yamanaka H., Fukuzawa M. (2010). Increased expression of the hedgehog signaling pathway in pediatric solid malignancies. J. Pediatr. Surg..

[26] Zweidler-McKay P.A. (2008). Notch signaling in pediatric malignancies. Curr. Oncol. Rep..

[27] Ahmed A.A., Mohamed A.D., Gener M., Li W., Taboada E. (2017). YAP and the Hippo pathway in pediatric cancer. Mol. Cell. Oncol..

[28] Strasser A., Jost P.J., Nagata S. (2009). The many roles of FAS receptor signaling in the immune system. Immunity.

[29] Tong N., Zhang L., Sheng X., Wang M., Zhang Z., Fang Y., Xue Y., Li J., Zhang Z. (2012). Functional polymorphisms in FAS, FASL and CASP8 genes and risk of childhood acute lymphoblastic leukemia: A case-control study. Leuk. Lymphoma.

[30] Komada Y., Zhou Y.W., Zhang X.L., Xue H.L., Sakai H., Tanaka S., Sakatoku H., Sakurai M. (1995). Fas receptor (CD95)-mediated apoptosis is induced in leukemic cells entering G1B compartment of the cell cycle. Blood.

[31] Plumas J., Jacob M.C., Chaperot L., Molens J.P., Sotto J.J., Bensa J.C. (1998). Tumor B cells from non-Hodgkin’s lymphoma are resistant to CD95 (Fas/Apo-1)-mediated apoptosis. Blood.

[32] Friesen C., Herr I., Krammer P.H., Debatin K.M. (1996). Involvement of the CD95 (APO-1/FAS) receptor/ligand system in drug-induced apoptosis in leukemia cells. Nat. Med..

[33] Friesen C., Fulda S., Debatin K.M. (1997). Deficient activation of the CD95 (APO-1/Fas) system in drug-resistant cells. Leukemia.

[34] Fulda S., Los M., Friesen C., Debatin K.M. (1998). Chemosensitivity of solid tumor cells in vitro is related to activation of the CD95 system. Int. J. Cancer.

[35] Nguyen P.L., Harris N.L., Ritz J., Robertson M.J. (1996). Expression of CD95 antigen and Bcl-2 protein in non-Hodgkin’s lymphomas and Hodgkin’s disease. Am. J. Pathol..

[36] Min Y.H., Lee S., Lee J.W., Chong S.Y., Hahn J.S., Ko Y.W. (1996). Expression of Fas antigen in acute myeloid leukaemia is associated with therapeutic response to chemotherapy. Br. J. Haematol..

[37] Friesen C., Fulda S., Debatin K.M. (1999). Cytotoxic drugs and the CD95 pathway. Leukemia.

[38] Debatin K.M., Krammer P.H. (1995). Resistance to APO-1 (CD95) induced apoptosis in T-ALL is determined by a BCL-2 independent anti-apoptotic program. Leukemia.

[39] Karawajew L., Wuchter C., Ruppert V., Drexler H., Gruss H.J., Dorken B., Ludwig W.D. (1997). Differential CD95 expression and function in T and B lineage acute lymphoblastic leukemia cells. Leukemia.

[40] Lucking-Famira K.M., Daniel P.T., Moller P., Krammer P.H., Debatin K.M. (1994). APO-1 (CD95) mediated apoptosis in human T-ALL engrafted in SCID mice. Leukemia.

[41] Cheng J., Zhou T., Liu C., Shapiro J.P., Brauer M.J., Kiefer M.C., Barr P.J., Mountz J.D. (1994). Protection from Fas-mediated apoptosis by a soluble form of the Fas molecule. Science.

[42] Wood C.M., Goodman P.A., Vassilev A.O., Uckun F.M. (2003). CD95 (APO-1/FAS) deficiency in infant acute lymphoblastic leukemia: Detection of novel soluble Fas splice variants. Eur. J. Haematol..

[43] Knipping E., Debatin K.M., Stricker K., Heilig B., Eder A., Krammer P.H. (1995). Identification of soluble APO-1 in supernatants of human B- and T-cell lines and increased serum levels in B- and T-cell leukemias. Blood.

[44] Munker R., Midis G., Owen-Schaub L., Andreff M. (1996). Soluble FAS (CD95) is not elevated in the serum of patients with myeloid leukemias, myeloproliferative and myelodysplastic syndromes. Leukemia.

[45] Cascino I., Papoff G., De Maria R., Testi R., Ruberti G. (1996). Fas/Apo-1 (CD95) receptor lacking the intracytoplasmic signaling domain protects tumor cells from Fas-mediated apoptosis. J. Immunol..

[46] Beltinger C., Kurz E., Bohler T., Schrappe M., Ludwig W.D., Debatin K.M. (1998). CD95 (APO-1/Fas) mutations in childhood T-lineage acute lymphoblastic leukemia. Blood.

[47] Sibley K., Rollinson S., Allan J.M., Smith A.G., Law G.R., Roddam P.L., Skibola C.F., Smith M.T., Morgan G.J. (2003). Functional FAS promoter polymorphisms are associated with increased risk of acute myeloid leukemia. Cancer Res..

[48] Muschen M., Rajewsky K., Kronke M., Kuppers R. (2002). The origin of CD95-gene mutations in B-cell lymphoma. Trends Immunol..

[49] Bazzoni F., Beutler B. (1996). The tumor necrosis factor ligand and receptor families. N. Engl. J. Med..

[50] Ogasawara J., Watanabe-Fukunaga R., Adachi M., Matsuzawa A., Kasugai T., Kitamura Y., Itoh N., Suda T., Nagata S. (1993). Lethal effect of the anti-Fas antibody in mice. Nature.

[51] Ashkenazi A., Pai R.C., Fong S., Leung S., Lawrence D.A., Marsters S.A., Blackie C., Chang L., McMurtrey A.E., Hebert A. (1999). Safety and antitumor activity of recombinant soluble Apo2 ligand. J. Clin. Investig..

[52] Ehrhardt H., Fulda S., Schmid I., Hiscott J., Debatin K.M., Jeremias I. (2003). TRAIL induced survival and proliferation in cancer cells resistant towards TRAIL-induced apoptosis mediated by NF-kappaB. Oncogene.

[53] Kontny H.U., Hammerle K., Klein R., Shayan P., Mackall C.L., Niemeyer C.M. (2001). Sensitivity of Ewing’s sarcoma to TRAIL-induced apoptosis. Cell Death Differ..

[54] Van Valen F., Fulda S., Truckenbrod B., Eckervogt V., Sonnemann J., Hillmann A., Rodl R., Hoffmann C., Winkelmann W., Schafer L. (2000). Apoptotic responsiveness of the Ewing’s sarcoma family of tumours to tumour necrosis factor-related apoptosis-inducing ligand (TRAIL). Int. J. Cancer.

[55] Petak I., Douglas L., Tillman D.M., Vernes R., Houghton J.A. (2000). Pediatric rhabdomyosarcoma cell lines are resistant to Fas-induced apoptosis and highly sensitive to TRAIL-induced apoptosis. Clin. Cancer Res..

[56] Yang X., Thiele C.J. (2003). Targeting the tumor necrosis factor-related apoptosis-inducing ligand path in neuroblastoma. Cancer Lett..

[57] Van Noesel M.M., Van Bezouw S., Voute P.A., Herman J.G., Pieters R., Versteeg R. (2003). Clustering of hypermethylated genes in neuroblastoma. Genes Chromosomes Cancer.

[58] Van Noesel M.M., van Bezouw S., Salomons G.S., Voute P.A., Pieters R., Baylin S.B., Herman J.G., Versteeg R. (2002). Tumor-specific down-regulation of the tumor necrosis factor-related apoptosis-inducing ligand decoy receptors DcR1 and DcR2 is associated with dense promoter hypermethylation. Cancer Res..

[59] Fulda S., Kufer M.U., Meyer E., van Valen F., Dockhorn-Dworniczak B., Debatin K.M. (2001). Sensitization for death receptor- or drug-induced apoptosis by re-expression of caspase-8 through demethylation or gene transfer. Oncogene.

[60] Yang Q., Kiernan C.M., Tian Y., Salwen H.R., Chlenski A., Brumback B.A., London W.B., Cohn S.L. (2007). Methylation of CASP8, DCR2, and HIN-1 in neuroblastoma is associated with poor outcome. Clin. Cancer Res..

[61] Teitz T., Wei T., Valentine M.B., Vanin E.F., Grenet J., Valentine V.A., Behm F.G., Look A.T., Lahti J.M., Kidd V.J. (2000). Caspase 8 is deleted or silenced preferentially in childhood neuroblastomas with amplification of MYCN. Nat. Med..

[62] Fakler M., Loeder S., Vogler M., Schneider K., Jeremias I., Debatin K.M., Fulda S. (2009). Small molecule XIAP inhibitors cooperate with TRAIL to induce apoptosis in childhood acute leukemia cells and overcome Bcl-2-mediated resistance. Blood.

[63] Micheau O., Shirley S., Dufour F. (2013). Death receptors as targets in cancer. Br. J. Pharmacol..

[64] Ashkenazi A., Herbst R.S. (2008). To kill a tumor cell: The potential of proapoptotic receptor agonists. J. Clin. Investig..

[65] Smith M.A., Morton C.L., Kolb E.A., Gorlick R., Keir S.T., Carol H., Lock R., Kang M.H., Reynolds C.P., Maris J.M. (2010). Initial testing (stage 1) of mapatumumab (HGS-ETR1) by the pediatric preclinical testing program. Pediatr. Blood Cancer.

[66] Merchant M.S., Geller J.I., Baird K., Chou A.J., Galli S., Charles A., Amaoko M., Rhee E.H., Price A., Wexler L.H. (2012). Phase I trial and pharmacokinetic study of lexatumumab in pediatric patients with solid tumors. J. Clin. Oncol..

[67] Gross A., McDonnell J.M., Korsmeyer S.J. (1999). BCL-2 family members and the mitochondria in apoptosis. Genes Dev..

[68] Vitagliano O., Addeo R., D’Angelo V., Indolfi C., Indolfi P., Casale F. (2013). The Bcl-2/Bax and Ras/Raf/MEK/ERK signaling pathways: Implications in pediatric leukemia pathogenesis and new prospects for therapeutic approaches. Expert Rev. Hematol..

[69] Wei C.C., Ball S., Lin L., Liu A., Fuchs J.R., Li P.K., Li C., Lin J. (2011). Two small molecule compounds, LLL12 and FLLL32, exhibit potent inhibitory activity on STAT3 in human rhabdomyosarcoma cells. Int. J. Oncol..

[70] Chang F., Lee J.T., Navolanic P.M., Steelman L.S., Shelton J.G., Blalock W.L., Franklin R.A., McCubrey J.A. (2003). Involvement of PI3K/Akt pathway in cell cycle progression, apoptosis, and neoplastic transformation: A target for cancer chemotherapy. Leukemia.

[71] Levine A.J., Momand J., Finlay C.A. (1991). The p53 tumour suppressor gene. Nature.

[72] Goldsmith K.C., Lestini B.J., Gross M., Ip L., Bhumbla A., Zhang X., Zhao H., Liu X., Hogarty M.D. (2010). BH3 response profiles from neuroblastoma mitochondria predict activity of small molecule Bcl-2 family antagonists. Cell Death Differ..

[73] Porwit-MacDonald A., Ivory K., Wilkinson S., Wheatley K., Wong L., Janossy G. (1995). Bcl-2 protein expression in normal human bone marrow precursors and in acute myelogenous leukemia. Leukemia.

[74] Banker D.E., Radich J., Becker A., Kerkof K., Norwood T., Willman C., Appelbaum F.R. (1998). The t(8;21) translocation is not consistently associated with high Bcl-2 expression in de novo acute myeloid leukemias of adults. Clin. Cancer Res..

[75] Coustan-Smith E., Kitanaka A., Pui C.H., McNinch L., Evans W.E., Raimondi S.C., Behm F.G., Arico M., Campana D. (1996). Clinical relevance of BCL-2 overexpression in childhood acute lymphoblastic leukemia. Blood.

[76] Campana D., Coustan-Smith E., Manabe A., Buschle M., Raimondi S.C., Behm F.G., Ashmun R., Arico M., Biondi A., Pui C.H. (1993). Prolonged survival of B-lineage acute lymphoblastic leukemia cells is accompanied by overexpression of bcl-2 protein. Blood.

[77] Prokop A., Wieder T., Sturm I., Essmann F., Seeger K., Wuchter C., Ludwig W.D., Henze G., Dorken B., Daniel P.T. (2000). Relapse in childhood acute lymphoblastic leukemia is associated with a decrease of the Bax/Bcl-2 ratio and loss of spontaneous caspase-3 processing in vivo. Leukemia.

[78] Del Poeta G., Venditti A., Del Principe M.I., Maurillo L., Buccisano F., Tamburini A., Cox M.C., Franchi A., Bruno A., Mazzone C. (2003). Amount of spontaneous apoptosis detected by Bax/Bcl-2 ratio predicts outcome in acute myeloid leukemia (AML). Blood.

[79] Nedelcu T., Kubista B., Koller A., Sulzbacher I., Mosberger I., Arrich F., Trieb K., Kotz R., Toma C.D. (2008). Livin and Bcl-2 expression in high-grade osteosarcoma. J. Cancer Res. Clin. Oncol..

[80] Ganigi P.M., Santosh V., Anandh B., Chandramouli B.A., Sastry Kolluri V.R. (2005). Expression of p53, EGFR, pRb and bcl-2 proteins in pediatric glioblastoma multiforme: A study of 54 patients. Pediatr. Neurosurg..

[81] Re G.G., Hazen-Martin D.J., El Bahtimi R., Brownlee N.A., Willingham M.C., Garvin A.J. (1999). Prognostic significance of Bcl-2 in Wilms’ tumor and oncogenic potential of Bcl-X(L) in rare tumor cases. Int. J. Cancer.

[82] Ikeda H., Hirato J., Akami M., Matsuyama S., Suzuki N., Takahashi A., Kuroiwa M. (1995). Bcl-2 oncoprotein expression and apoptosis in neuroblastoma. J. Pediatr. Surg..

[83] Lasorella A., Iavarone A., Israel M.A. (1995). Differentiation of neuroblastoma enhances Bcl-2 expression and induces alterations of apoptosis and drug resistance. Cancer Res..

[84] Hanada M., Krajewski S., Tanaka S., Cazals-Hatem D., Spengler B.A., Ross R.A., Biedler J.L., Reed J.C. (1993). Regulation of Bcl-2 oncoprotein levels with differentiation of human neuroblastoma cells. Cancer Res..

[85] Kirkin V., Joos S., Zornig M. (2004). The role of Bcl-2 family members in tumorigenesis. Biochim. Biophys. Acta.

[86] Ganjavi H., Gee M., Narendran A., Parkinson N., Krishnamoorthy M., Freedman M.H., Malkin D. (2006). Adenovirus-mediated p53 gene therapy in osteosarcoma cell lines: Sensitization to cisplatin and doxorubicin. Cancer Gene Ther..

[87] Ganjavi H., Gee M., Narendran A., Freedman M.H., Malkin D. (2005). Adenovirus-mediated p53 gene therapy in pediatric soft-tissue sarcoma cell lines: Sensitization to cisplatin and doxorubicin. Cancer Gene Ther..

[88] Shetty S., Taylor A.C., Harris L.C. (2002). Selective chemosensitization of rhabdomyosarcoma cell lines following wild-type p53 adenoviral transduction. Anticancer Drugs.

[89] Cai B., Chang S.H., Becker E.B., Bonni A., Xia Z. (2006). p38 MAP kinase mediates apoptosis through phosphorylation of BimEL at Ser-65. J. Biol. Chem..

[90] Lu J., Quearry B., Harada H. (2006). p38-MAP kinase activation followed by BIM induction is essential for glucocorticoid-induced apoptosis in lymphoblastic leukemia cells. FEBS Lett..

[91] Shimo T., Matsumura S., Ibaragi S., Isowa S., Kishimoto K., Mese H., Nishiyama A., Sasaki A. (2007). Specific inhibitor of MEK-mediated cross-talk between ERK and p38 MAPK during differentiation of human osteosarcoma cells. J. Cell Commun. Signal..

[92] Nowicki M., Miskowiak B. (2002). Comparison of the cell immunophenotype of metastatic and primary foci in stage IV-S neuroblastoma. Folia Histochem. Cytobiol..

[93] Nowicki M., Miskowiak B., Ostalska-Nowicka D. (2003). Detection of substance P and its mRNA in human blast cells in childhood lymphoblastic leukaemia using immunocytochemistry and in situ hybridisation. Folia Histochem. Cytobiol..

[94] DeFea K.A., Vaughn Z.D., O’Bryan E.M., Nishijima D., Dery O., Bunnett N.W. (2000). The proliferative and antiapoptotic effects of substance P are facilitated by formation of a beta -arrestin-dependent scaffolding complex. Proc. Natl. Acad. Sci. USA.

[95] Munoz M., Covenas R. (2013). Involvement of substance P and the NK-1 receptor in cancer progression. Peptides.

[96] Bender A., Opel D., Naumann I., Kappler R., Friedman L., von Schweinitz D., Debatin K.M., Fulda S. (2011). PI3K inhibitors prime neuroblastoma cells for chemotherapy by shifting the balance towards pro-apoptotic Bcl-2 proteins and enhanced mitochondrial apoptosis. Oncogene.

[97] Toretsky J.A., Thakar M., Eskenazi A.E., Frantz C.N. (1999). Phosphoinositide 3-hydroxide kinase blockade enhances apoptosis in the Ewing’s sarcoma family of tumors. Cancer Res..

[98] Eichenmuller M., Gruner I., Hagl B., Haberle B., Muller-Hocker J., Von Schweinitz D., Kappler R. (2009). Blocking the hedgehog pathway inhibits hepatoblastoma growth. Hepatology.

[99] Oliver T.G., Grasfeder L.L., Carroll A.L., Kaiser C., Gillingham C.L., Lin S.M., Wickramasinghe R., Scott M.P., Wechsler-Reya R.J. (2003). Transcriptional profiling of the Sonic hedgehog response: A critical role for N-myc in proliferation of neuronal precursors. Proc. Natl. Acad. Sci. USA.

[100] Teglund S., Toftgard R. (2010). Hedgehog beyond medulloblastoma and basal cell carcinoma. Biochim. Biophys. Acta.

[101] Pressey J.G., Anderson J.R., Crossman D.K., Lynch J.C., Barr F.G. (2011). Hedgehog pathway activity in pediatric embryonal rhabdomyosarcoma and undifferentiated sarcoma: A report from the Children’s Oncology Group. Pediatr. Blood Cancer.

[102] Bigelow R.L., Chari N.S., Unden A.B., Spurgers K.B., Lee S., Roop D.R., Toftgard R., McDonnell T.J. (2004). Transcriptional regulation of bcl-2 mediated by the sonic hedgehog signaling pathway through gli-1. J. Biol. Chem..

[103] Bar E.E., Chaudhry A., Farah M.H., Eberhart C.G. (2007). Hedgehog signaling promotes medulloblastoma survival via Bc/II. Am. J. Pathol..

[104] Baliko F., Bright T., Poon R., Cohen B., Egan S.E., Alman B.A. (2007). Inhibition of notch signaling induces neural differentiation in Ewing sarcoma. Am. J. Pathol..

[105] Zhang P., Yang Y., Zweidler-McKay P.A., Hughes D.P. (2008). Critical role of notch signaling in osteosarcoma invasion and metastasis. Clin. Cancer Res..

[106] Fan X., Eberhart C.G. (2008). Medulloblastoma stem cells. J. Clin. Oncol..

[107] Aster J.C., Pear W.S., Blacklow S.C. (2008). Notch signaling in leukemia. Annu. Rev. Pathol..

[108] Zhang H., Cai X., Wang Y., Tang H., Tong D., Ji F. (2010). microRNA-143, down-regulated in osteosarcoma, promotes apoptosis and suppresses tumorigenicity by targeting Bcl-2. Oncol. Rep..

[109] Margue C.M., Bernasconi M., Barr F.G., Schafer B.W. (2000). Transcriptional modulation of the anti-apoptotic protein BCL-XL by the paired box transcription factors PAX3 and PAX3/FKHR. Oncogene.

[110] Liu A., Liu Y., Xu Z., Yu W., Wang H., Li C., Lin J. (2011). Novel small molecule, XZH-5, inhibits constitutive and interleukin-6-induced STAT3 phosphorylation in human rhabdomyosarcoma cells. Cancer Sci..

[111] McPake C.R., Tillman D.M., Poquette C.A., George E.O., Houghton J.A., Harris L.C. (1998). Bax is an important determinant of chemosensitivity in pediatric tumor cell lines independent of Bcl-2 expression and p53 status. Oncol. Res..

[112] Fulda S., Meyer E., Debatin K.M. (2002). Inhibition of TRAIL-induced apoptosis by Bcl-2 overexpression. Oncogene.

[113] Dole M., Nunez G., Merchant A.K., Maybaum J., Rode C.K., Bloch C.A., Castle V.P. (1994). Bcl-2 inhibits chemotherapy-induced apoptosis in neuroblastoma. Cancer Res..

[114] Dole M.G., Jasty R., Cooper M.J., Thompson C.B., Nunez G., Castle V.P. (1995). Bcl-xL is expressed in neuroblastoma cells and modulates chemotherapy-induced apoptosis. Cancer Res..

[115] Campos L., Rouault J.P., Sabido O., Oriol P., Roubi N., Vasselon C., Archimbaud E., Magaud J.P., Guyotat D. (1993). High expression of bcl-2 protein in acute myeloid leukemia cells is associated with poor response to chemotherapy. Blood.

[116] Oltersdorf T., Elmore S.W., Shoemaker A.R., Armstrong R.C., Augeri D.J., Belli B.A., Bruncko M., Deckwerth T.L., Dinges J., Hajduk P.J. (2005). An inhibitor of Bcl-2 family proteins induces regression of solid tumours. Nature.

[117] Del Gaizo Moore V., Schlis K.D., Sallan S.E., Armstrong S.A., Letai A. (2008). BCL-2 dependence and ABT-737 sensitivity in acute lymphoblastic leukemia. Blood.

[118] Kang M.H., Kang Y.H., Szymanska B., Wilczynska-Kalak U., Sheard M.A., Harned T.M., Lock R.B., Reynolds C.P. (2007). Activity of vincristine, L-ASP, and dexamethasone against acute lymphoblastic leukemia is enhanced by the BH3-mimetic ABT-737 in vitro and in vivo. Blood.

[119] High L.M., Szymanska B., Wilczynska-Kalak U., Barber N., O’Brien R., Khaw S.L., Vikstrom I.B., Roberts A.W., Lock R.B. (2010). The Bcl-2 homology domain 3 mimetic ABT-737 targets the apoptotic machinery in acute lymphoblastic leukemia resulting in synergistic in vitro and in vivo interactions with established drugs. Mol. Pharmacol..

[120] Goldsmith K.C., Gross M., Peirce S., Luyindula D., Liu X., Vu A., Sliozberg M., Guo R., Zhao H., Reynolds C.P. (2012). Mitochondrial Bcl-2 family dynamics define therapy response and resistance in neuroblastoma. Cancer Res..

[121] Lin X., Morgan-Lappe S., Huang X., Li L., Zakula D.M., Vernetti L.A., Fesik S.W., Shen Y. (2007). ‘Seed’ analysis of off-target siRNAs reveals an essential role of Mcl-1 in resistance to the small-molecule Bcl-2/Bcl-XL inhibitor ABT-737. Oncogene.

[122] Chen S., Dai Y., Harada H., Dent P., Grant S. (2007). Mcl-1 down-regulation potentiates ABT-737 lethality by cooperatively inducing Bak activation and Bax translocation. Cancer Res..

[123] Fang H., Harned T.M., Kalous O., Maldonado V., DeClerck Y.A., Reynolds C.P. (2011). Synergistic activity of fenretinide and the Bcl-2 family protein inhibitor ABT-737 against human neuroblastoma. Clin. Cancer Res..

[124] Lestini B.J., Goldsmith K.C., Fluchel M.N., Liu X., Chen N.L., Goyal B., Pawel B.R., Hogarty M.D. (2009). Mcl1 downregulation sensitizes neuroblastoma to cytotoxic chemotherapy and small molecule Bcl2-family antagonists. Cancer Biol. Ther..

[125] Preuss E., Hugle M., Reimann R., Schlecht M., Fulda S. (2013). Pan-mammalian target of rapamycin (mTOR) inhibitor AZD8055 primes rhabdomyosarcoma cells for ABT-737-induced apoptosis by down-regulating Mcl-1 protein. J. Biol. Chem..

[126] Kang M.H., Wan Z., Kang Y.H., Sposto R., Reynolds C.P. (2008). Mechanism of synergy of N-(4-hydroxyphenyl)retinamide and ABT-737 in acute lymphoblastic leukemia cell lines: Mcl-1 inactivation. J. Natl. Cancer Inst..

[127] Shore G.C., Viallet J. (2005). Modulating the bcl-2 family of apoptosis suppressors for potential therapeutic benefit in cancer. Hematol. Am. Soc. Hematol. Educ. Program.

[128] Konopleva M., Contractor R., Tsao T., Samudio I., Ruvolo P.P., Kitada S., Deng X., Zhai D., Shi Y.X., Sneed T. (2006). Mechanisms of apoptosis sensitivity and resistance to the BH3 mimetic ABT-737 in acute myeloid leukemia. Cancer Cell.

[129] Kojima K., Konopleva M., Samudio I.J., Schober W.D., Bornmann W.G., Andreeff M. (2006). Concomitant inhibition of MDM2 and Bcl-2 protein function synergistically induce mitochondrial apoptosis in AML. Cell Cycle.

[130] Van Delft M.F., Wei A.H., Mason K.D., Vandenberg C.J., Chen L., Czabotar P.E., Willis S.N., Scott C.L., Day C.L., Cory S. (2006). The BH3 mimetic ABT-737 targets selective Bcl-2 proteins and efficiently induces apoptosis via Bak/Bax if Mcl-1 is neutralized. Cancer Cell.

[131] Tse C., Shoemaker A.R., Adickes J., Anderson M.G., Chen J., Jin S., Johnson E.F., Marsh K.C., Mitten M.J., Nimmer P. (2008). ABT-263: A potent and orally bioavailable Bcl-2 family inhibitor. Cancer Res..

[132] Lock R., Carol H., Houghton P.J., Morton C.L., Kolb E.A., Gorlick R., Reynolds C.P., Maris J.M., Keir S.T., Wu J. (2008). Initial testing (stage 1) of the BH3 mimetic ABT-263 by the pediatric preclinical testing program. Pediatr. Blood Cancer.

[133] Suryani S., Carol H., Chonghaile T.N., Frismantas V., Sarmah C., High L., Bornhauser B., Cowley M.J., Szymanska B., Evans K. (2014). Cell and molecular determinants of in vivo efficacy of the BH3 mimetic ABT-263 against pediatric acute lymphoblastic leukemia xenografts. Clin. Cancer Res..

[134] Faqar-Uz-Zaman S.F., Heinicke U., Meister M.T., Vogler M., Fulda S. (2018). BCL-xL-selective BH3 mimetic sensitizes rhabdomyosarcoma cells to chemotherapeutics by activation of the mitochondrial pathway of apoptosis. Cancer Lett..

[135] Klenke S., Akdeli N., Stelmach P., Heukamp L., Schulte J.H., Bachmann H.S. (2019). The small molecule Bcl-2/Mcl-1 inhibitor TW-37 shows single-agent cytotoxicity in neuroblastoma cell lines. BMC Cancer.

[136] Rheingold S.R., Hogarty M.D., Blaney S.M., Zwiebel J.A., Sauk-Schubert C., Chandula R., Krailo M.D., Adamson P.C., Children’s Oncology Group S. (2007). Phase I Trial of G3139, a bcl-2 antisense oligonucleotide, combined with doxorubicin and cyclophosphamide in children with relapsed solid tumors: A Children’s Oncology Group Study. J. Clin. Oncol..

[137] Marzo I., Naval J. (2008). Bcl-2 family members as molecular targets in cancer therapy. Biochem. Pharmacol..

[138] Salvesen G.S., Duckett C.S. (2002). IAP proteins: Blocking the road to death’s door. Nat. Rev. Mol. Cell Biol..

[139] Wei Y., Fan T., Yu M. (2008). Inhibitor of apoptosis proteins and apoptosis. Acta Biochim. Biophys. Sin..

[140] Sung K.W., Choi J., Hwang Y.K., Lee S.J., Kim H.J., Kim J.Y., Cho E.J., Yoo K.H., Koo H.H. (2009). Overexpression of X-linked inhibitor of apoptosis protein (XIAP) is an independent unfavorable prognostic factor in childhood de novo acute myeloid leukemia. J. Korean Med. Sci..

[141] Tamm I., Richter S., Oltersdorf D., Creutzig U., Harbott J., Scholz F., Karawajew L., Ludwig W.D., Wuchter C. (2004). High expression levels of x-linked inhibitor of apoptosis protein and survivin correlate with poor overall survival in childhood de novo acute myeloid leukemia. Clin. Cancer Res..

[142] Hundsdoerfer P., Dietrich I., Schmelz K., Eckert C., Henze G. (2010). XIAP expression is post-transcriptionally upregulated in childhood ALL and is associated with glucocorticoid response in T-cell ALL. Pediatr. Blood Cancer.

[143] Holcik M., Lefebvre C., Yeh C., Chow T., Korneluk R.G. (1999). A new internal-ribosome-entry-site motif potentiates XIAP-mediated cytoprotection. Nat. Cell Biol..

[144] Saraei R., Soleimani M., Movassaghpour Akbari A.A., Farshdousti Hagh M., Hassanzadeh A., Solali S. (2018). The role of XIAP in resistance to TNF-related apoptosis-inducing ligand (TRAIL) in Leukemia. Biomed. Pharmacother..

[145] Altieri D.C. (2003). Validating survivin as a cancer therapeutic target. Nat. Rev. Cancer.

[146] Jiang Y., de Bruin A., Caldas H., Fangusaro J., Hayes J., Conway E.M., Robinson M.L., Altura R.A. (2005). Essential role for survivin in early brain development. J. Neurosci..

[147] Xing Z., Conway E.M., Kang C., Winoto A. (2004). Essential role of survivin, an inhibitor of apoptosis protein, in T cell development, maturation, and homeostasis. J. Exp. Med..

[148] O’Driscoll L., Linehan R., Clynes M. (2003). Survivin: Role in normal cells and in pathological conditions. Curr. Cancer Drug Targets.

[149] Bai H., Gayyed M.F., Lam-Himlin D.M., Klein A.P., Nayar S.K., Xu Y., Khan M., Argani P., Pan D., Anders R.A. (2012). Expression of Yes-associated protein modulates Survivin expression in primary liver malignancies. Hum. Pathol..

[150] Cottini F., Anderson K.C., Tonon G. (2014). Awakening the Hippo co-activator YAP1, a mercurial cancer gene, in hematologic cancers. Mol. Cell. Oncol..

[151] Bouvier C., Macagno N., Nguyen Q., Loundou A., Jiguet-Jiglaire C., Gentet J.C., Jouve J.L., Rochwerger A., Mattei J.C., Bouvard D. (2016). Prognostic value of the Hippo pathway transcriptional coactivators YAP/TAZ and beta1-integrin in conventional osteosarcoma. Oncotarget.

[152] Ahmed A.A., Abedalthagafi M., Anwar A.E., Bui M.M. (2015). Akt and hippo pathways in Ewing’s sarcoma tumors and their prognostic significance. J. Cancer.

[153] Slemmons K.K., Crose L.E., Rudzinski E., Bentley R.C., Linardic C.M. (2015). Role of the YAP Oncoprotein in Priming Ras-Driven Rhabdomyosarcoma. PLoS ONE.

[154] Moore A.S., Alonzo T.A., Gerbing R.B., Lange B.J., Heerema N.A., Franklin J., Raimondi S.C., Hirsch B.A., Gamis A.S., Meshinchi S. (2014). BIRC5 (survivin) splice variant expression correlates with refractory disease and poor outcome in pediatric acute myeloid leukemia: A report from the Children’s Oncology Group. Pediatr. Blood Cancer.

[155] Troeger A., Siepermann M., Escherich G., Meisel R., Willers R., Gudowius S., Moritz T., Laws H.J., Hanenberg H., Goebel U. (2007). Survivin and its prognostic significance in pediatric acute B-cell precursor lymphoblastic leukemia. Haematologica.

[156] Islam A., Kageyama H., Takada N., Kawamoto T., Takayasu H., Isogai E., Ohira M., Hashizume K., Kobayashi H., Kaneko Y. (2000). High expression of Survivin, mapped to 17q25, is significantly associated with poor prognostic factors and promotes cell survival in human neuroblastoma. Oncogene.

[157] Takamizawa S., Scott D., Wen J., Grundy P., Bishop W., Kimura K., Sandler A. (2001). The survivin:fas ratio in pediatric renal tumors. J. Pediatr. Surg..

[158] Zou J., Gan M., Mao N., Zhu X., Shi Q., Yang H. (2010). Sensitization of osteosarcoma cell line SaOS-2 to chemotherapy by downregulating survivin. Arch. Med. Res..

[159] Caldas H., Holloway M.P., Hall B.M., Qualman S.J., Altura R.A. (2006). Survivin-directed RNA interference cocktail is a potent suppressor of tumour growth in vivo. J. Med. Genet..

[160] Hingorani P., Dickman P., Garcia-Filion P., White-Collins A., Kolb E.A., Azorsa D.O. (2013). BIRC5 expression is a poor prognostic marker in Ewing sarcoma. Pediatr. Blood Cancer.

[161] Ito R., Asami S., Motohashi S., Ootsuka S., Yamaguchi Y., Chin M., Shichino H., Yoshida Y., Nemoto N., Mugishima H. (2005). Significance of survivin mRNA expression in prognosis of neuroblastoma. Biol. Pharm. Bull..

[162] Miller M.A., Ohashi K., Zhu X., McGrady P., London W.B., Hogarty M., Sandler A.D. (2006). Survivin mRNA levels are associated with biology of disease and patient survival in neuroblastoma: A report from the children’s oncology group. J. Pediatr. Hematol. Oncol..

[163] Adida C., Berrebi D., Peuchmaur M., Reyes-Mugica M., Altieri D.C. (1998). Anti-apoptosis gene, survivin, and prognosis of neuroblastoma. Lancet.

[164] Azuhata T., Scott D., Takamizawa S., Wen J., Davidoff A., Fukuzawa M., Sandler A. (2001). The inhibitor of apoptosis protein survivin is associated with high-risk behavior of neuroblastoma. J. Pediatr. Surg..

[165] Li F. (2005). Role of survivin and its splice variants in tumorigenesis. Br. J. Cancer.

[166] Troger A., Siepermann M., Mahotka C., Wethkamp N., Bulle H., Laws H.J., Escherich G., Janka-Schaub G., Gobel U., Dilloo D. (2007). Role of survivin splice variants in pediatric acute precursor B lymphoblastic leukemia. Klin. Padiatr..

[167] Trieb K., Lehner R., Stulnig T., Sulzbacher I., Shroyer K.R. (2003). Survivin expression in human osteosarcoma is a marker for survival. Eur. J. Surg. Oncol..

[168] Sung K.W., Choi J., Hwang Y.K., Lee S.J., Kim H.J., Lee S.H., Yoo K.H., Jung H.L., Koo H.H. (2007). Overexpression of Apollon, an antiapoptotic protein, is associated with poor prognosis in childhood de novo acute myeloid leukemia. Clin. Cancer Res..

[169] Ismail E.A., Mahmoud H.M., Tawfik L.M., Habashy D.M., Adly A.A., El-Sherif N.H., Abdelwahab M.A. (2012). BIRC6/Apollon gene expression in childhood acute leukemia: Impact on therapeutic response and prognosis. Eur. J. Haematol..

[170] Ibrahim L., Aladle D., Mansour A., Hammad A., Al Wakeel A.A., Abd El-Hameed S.A. (2014). Expression and prognostic significance of livin/BIRC7 in childhood acute lymphoblastic leukemia. Med. Oncol..

[171] Choi J., Hwang Y.K., Sung K.W., Lee S.H., Yoo K.H., Jung H.L., Koo H.H., Kim H.J., Kang H.J., Shin H.Y. (2007). Expression of Livin, an antiapoptotic protein, is an independent favorable prognostic factor in childhood acute lymphoblastic leukemia. Blood.

[172] Nachmias B., Ashhab Y., Bucholtz V., Drize O., Kadouri L., Lotem M., Peretz T., Mandelboim O., Ben-Yehuda D. (2003). Caspase-mediated cleavage converts Livin from an antiapoptotic to a proapoptotic factor: Implications for drug-resistant melanoma. Cancer Res..

[173] Chen D.J., Huerta S. (2009). Smac mimetics as new cancer therapeutics. Anticancer Drugs.

[174] Pluta A., Wrzesien-Kus A., Cebula-Obrzut B., Wolska A., Szmigielska-Kaplon A., Czemerska M., Pluta P., Robak T., Smolewski P., Wierzbowska A. (2010). Influence of high expression of Smac/DIABLO protein on the clinical outcome in acute myeloid leukemia patients. Leuk. Res..

[175] Hotta T., Suzuki H., Nagai S., Yamamoto K., Imakiire A., Takada E., Itoh M., Mizuguchi J. (2003). Chemotherapeutic agents sensitize sarcoma cell lines to tumor necrosis factor-related apoptosis-inducing ligand-induced caspase-8 activation, apoptosis and loss of mitochondrial membrane potential. J. Orthop. Res..

[176] Arora V., Cheung H.H., Plenchette S., Micali O.C., Liston P., Korneluk R.G. (2007). Degradation of survivin by the X-linked inhibitor of apoptosis (XIAP)-XAF1 complex. J. Biol. Chem..

[177] Chen Z.X., Wallis K., Fell S.M., Sobrado V.R., Hemmer M.C., Ramskold D., Hellman U., Sandberg R., Kenchappa R.S., Martinson T. (2014). RNA helicase A is a downstream mediator of KIF1Bbeta tumor-suppressor function in neuroblastoma. Cancer Discov..

[178] Wright K.M., Vaughn A.E., Deshmukh M. (2007). Apoptosome dependent caspase-3 activation pathway is non-redundant and necessary for apoptosis in sympathetic neurons. Cell Death Differ..

[179] Deshmukh M., Johnson E.M. (1998). Evidence of a novel event during neuronal death: Development of competence-to-die in response to cytoplasmic cytochrome c. Neuron.

[180] Edwards S.N., Tolkovsky A.M. (1994). Characterization of apoptosis in cultured rat sympathetic neurons after nerve growth factor withdrawal. J. Cell Biol..

[181] Deckwerth T.L., Johnson E.M. (1993). Temporal analysis of events associated with programmed cell death (apoptosis) of sympathetic neurons deprived of nerve growth factor. J. Cell Biol..

[182] Schlisio S. (2009). Neuronal apoptosis by prolyl hydroxylation: Implication in nervous system tumours and the Warburg conundrum. J. Cell. Mol. Med..

[183] Zhu Y., Parada L.F. (2002). The molecular and genetic basis of neurological tumours. Nat. Rev. Cancer.

[184] Choo Z., Koh R.Y., Wallis K., Koh T.J., Kuick C.H., Sobrado V., Kenchappa R.S., Loh A.H., Soh S.Y., Schlisio S. (2016). XAF1 promotes neuroblastoma tumor suppression and is required for KIF1Bbeta-mediated apoptosis. Oncotarget.

[185] Varfolomeev E., Blankenship J.W., Wayson S.M., Fedorova A.V., Kayagaki N., Garg P., Zobel K., Dynek J.N., Elliott L.O., Wallweber H.J. (2007). IAP antagonists induce autoubiquitination of c-IAPs, NF-kappaB activation, and TNFalpha-dependent apoptosis. Cell.

[186] Fulda S., Vucic D. (2012). Targeting IAP proteins for therapeutic intervention in cancer. Nat. Rev. Drug Discov..

[187] Langemann D., Trochimiuk M., Appl B., Hundsdoerfer P., Reinshagen K., Eschenburg G. (2017). Sensitization of neuroblastoma for vincristine-induced apoptosis by Smac mimetic LCL161 is attended by G2 cell cycle arrest but is independent of NFkappaB, RIP1 and TNF-alpha. Oncotarget.

[188] Eschenburg G., Eggert A., Schramm A., Lode H.N., Hundsdoerfer P. (2012). Smac mimetic LBW242 sensitizes XIAP-overexpressing neuroblastoma cells for TNF-alpha-independent apoptosis. Cancer Res..

[189] Fulda S., Wick W., Weller M., Debatin K.M. (2002). Smac agonists sensitize for Apo2L/TRAIL- or anticancer drug-induced apoptosis and induce regression of malignant glioma in vivo. Nat. Med..

[190] Giagkousiklidis S., Vogler M., Westhoff M.A., Kasperczyk H., Debatin K.M., Fulda S. (2005). Sensitization for gamma-irradiation-induced apoptosis by second mitochondria-derived activator of caspase. Cancer Res..

[191] Vogler M., Giagkousiklidis S., Genze F., Gschwend J.E., Debatin K.M., Fulda S. (2005). Inhibition of clonogenic tumor growth: A novel function of Smac contributing to its antitumor activity. Oncogene.

[192] Houghton P.J., Kang M.H., Reynolds C.P., Morton C.L., Kolb E.A., Gorlick R., Keir S.T., Carol H., Lock R., Maris J.M. (2012). Initial testing (stage 1) of LCL161, a SMAC mimetic, by the Pediatric Preclinical Testing Program. Pediatr. Blood Cancer.

[193] Schirmer M., Trentin L., Queudeville M., Seyfried F., Demir S., Tausch E., Stilgenbauer S., Eckhoff S.M., Meyer L.H., Debatin K.M. (2016). Intrinsic and chemo-sensitizing activity of SMAC-mimetics on high-risk childhood acute lymphoblastic leukemia. Cell Death Dis..

[194] Gerges S., Rohde K., Fulda S. (2016). Cotreatment with Smac mimetics and demethylating agents induces both apoptotic and necroptotic cell death pathways in acute lymphoblastic leukemia cells. Cancer Lett..

[195] LaCasse E.C., Cherton-Horvat G.G., Hewitt K.E., Jerome L.J., Morris S.J., Kandimalla E.R., Yu D., Wang H., Wang W., Zhang R. (2006). Preclinical characterization of AEG35156/GEM 640, a second-generation antisense oligonucleotide targeting X-linked inhibitor of apoptosis. Clin. Cancer Res..

[196] Lacasse E.C., Kandimalla E.R., Winocour P., Sullivan T., Agrawal S., Gillard J.W., Durkin J. (2005). Application of XIAP antisense to cancer and other proliferative disorders: Development of AEG35156/GEM640. Ann. NY Acad. Sci..

[197] Holt S.V., Brookes K.E., Dive C., Makin G.W. (2011). Down-regulation of XIAP by AEG35156 in paediatric tumour cells induces apoptosis and sensitises cells to cytotoxic agents. Oncol. Rep..

[198] Schimmer A.D., Estey E.H., Borthakur G., Carter B.Z., Schiller G.J., Tallman M.S., Altman J.K., Karp J.E., Kassis J., Hedley D.W. (2009). Phase I/II trial of AEG35156 X-linked inhibitor of apoptosis protein antisense oligonucleotide combined with idarubicin and cytarabine in patients with relapsed or primary refractory acute myeloid leukemia. J. Clin. Oncol..

[199] Loder S., Fakler M., Schoeneberger H., Cristofanon S., Leibacher J., Vanlangenakker N., Bertrand M.J., Vandenabeele P., Jeremias I., Debatin K.M. (2012). RIP1 is required for IAP inhibitor-mediated sensitization of childhood acute leukemia cells to chemotherapy-induced apoptosis. Leukemia.

[200] Loeder S., Drensek A., Jeremias I., Debatin K.M., Fulda S. (2010). Small molecule XIAP inhibitors sensitize childhood acute leukemia cells for CD95-induced apoptosis. Int. J. Cancer.

[201] Yang T., Lan J., Huang Q., Chen X., Sun X., Liu X., Yang P., Jin T., Wang S., Mou X. (2015). Embelin sensitizes acute myeloid leukemia cells to TRAIL through XIAP inhibition and NF-kappaB inactivation. Cell Biochem. Biophys..

[202] Moreno-Martinez D., Nomdedeu M., Lara-Castillo M.C., Etxabe A., Pratcorona M., Tesi N., Diaz-Beya M., Rozman M., Montserrat E., Urbano-Ispizua A. (2014). XIAP inhibitors induce differentiation and impair clonogenic capacity of acute myeloid leukemia stem cells. Oncotarget.

[203] Zhang S., Wang X., Gu Z., Wang L. (2016). Small molecule survivin inhibitor YM155 displays potent activity against human osteosarcoma cells. Cancer Investig..

[204] Schultze K., Bock B., Eckert A., Oevermann L., Ramacher D., Wiestler O., Roth W. (2006). Troglitazone sensitizes tumor cells to TRAIL-induced apoptosis via down-regulation of FLIP and Survivin. Apoptosis.

[205] Kim S., Kang J., Qiao J., Thomas R.P., Evers B.M., Chung D.H. (2004). Phosphatidylinositol 3-kinase inhibition down-regulates survivin and facilitates TRAIL-mediated apoptosis in neuroblastomas. J. Pediatr. Surg..

[206] Shankar S.L., Mani S., O’Guin K.N., Kandimalla E.R., Agrawal S., Shafit-Zagardo B. (2001). Survivin inhibition induces human neural tumor cell death through caspase-independent and -dependent pathways. J. Neurochem..

[207] Fest S., Huebener N., Bleeke M., Durmus T., Stermann A., Woehler A., Baykan B., Zenclussen A.C., Michalsky E., Jaeger I.S. (2009). Survivin minigene DNA vaccination is effective against neuroblastoma. Int. J. Cancer.

